# Mitophagy deficiency activates stimulator of interferon genes activation and aggravates pathogenetic cardiac remodeling

**DOI:** 10.1016/j.gendis.2023.08.003

**Published:** 2023-09-02

**Authors:** Guoxiang Zhou, Xiaowen Wang, Mingyu Guo, Can Qu, Lei Gao, Jiang Yu, Yuanjing Li, Suxin Luo, Qiong Shi, Yongzheng Guo

**Affiliations:** a Division of Cardiology, The First Affiliated Hospital of Chongqing Medical University, Chongqing 400016, China; b Department of Cardiothoracic Surgery, The First Affiliated Hospital of Chongqing Medical University, Chongqing 400016, China; c Department of Pharmacy, The First Affiliated Hospital of Chongqing Medical University, Chongqing 400016, China; d The Department of Laboratory Medicine, M.O.E. Key Laboratory of Laboratory Medical Diagnostics, Chongqing Medical University, Chongqing 400016, China

**Keywords:** Cardiac remodeling, Mitochondrial autophagy, mtDNA, Sterile inflammation, STING

## Abstract

Stimulator of interferon genes (STING) has recently been found to play a crucial role in cardiac sterile inflammation and dysfunction. The role of stimulator of interferon genes (STING) in cardiac sterile inflammation and dysfunction has been recently discovered. This study aims to examine the involvement of STING in pathological cardiac remodeling and the mechanisms that govern the activation of the STING pathway. To investigate this, transverse aortic constriction (TAC) was performed on STING knockout mice to induce pressure overload-induced cardiac remodeling. Subsequently, cardiac function, remodeling, and inflammation levels were evaluated. The STING pathway was found to be activated in the pressure overload-stressed heart and angiotensin II (Ang II)-stimulated cardiac fibroblasts. Loss of STING expression led to a significant reduction in inflammatory responses, mitochondrial fragmentation, and oxidative stress in the heart, resulting in attenuated cardiac remodeling and dysfunction. Furthermore, the exacerbation of pressure overload-induced STING-mediated inflammation and pathological cardiac remodeling was observed when mitophagy was suppressed through the silencing of Parkin, an E3 ubiquitin ligase. Taken together, these findings indicate that STING represents a newly identified and significant molecule implicated in the process of pathological cardiac remodeling and that mitophagy is an upstream mechanism that regulates STING activation. Targeting STING may therefore provide a novel therapeutic strategy for pathological cardiac remodeling and heart failure.

## Introduction

Despite improvements in the management of cardiovascular diseases, heart failure remains one of the leading causes of mortality and morbidity worldwide, indicating the need for new therapeutic strategies to prevent adverse outcomes. Cardiac remodeling, which is primarily characterized by interstitial fibrosis and hypertrophy, is a major cause of cardiac dysfunction.^1^ Cardiac fibroblasts (CFs) play a dominant role in the process of interstitial fibrosis by over-proliferating and producing excessive collagen and extracellular matrix.^2^ While infection is typically not linked to the occurrence of cardiac remodeling and heart failure, an increasing body of evidence suggests that inflammatory responses play a role in the deterioration of pump function.^3^^,^^4^ However, clinical trials focused on inflammation during heart failure have shown neutral effects or a worsened clinical outcome,^5^ suggesting a lack of understanding of the role of the immune system in the pathogenesis of heart failure. Thus, further studies on the molecular mechanisms initiating sterile inflammatory responses in the heart are warranted.

The cyclic GMP-AMP synthase (cGAS)-stimulator of interferon genes (STING) pathway is a newly discovered cytosolic DNA sensing pathway and is significantly involved into the initiation of sterile inflammation in the stressed heart.^6^, ^7^, ^8^ Once cGAS binds to cytosolic DNA, STING is activated and subsequently induces the phosphorylation and activation of its downstream target, interferon regulatory factor 3 (IRF3).^9^ Phospho-IRF3 (p-IRF3) then transports to the nucleus and promotes the transcription of IFN and IFN-stimulated nuclear genes (ISGs), leading to the induction of inflammation.^10^ However, the potential roles of STING in pressure overload-induced cardiac fibrosis and remodeling have not yet been fully investigated.

Mitochondria are abundant in the heart. In addition to their role in energy production, mitochondria also participate in the regulation of inflammatory responses.^11^ Due to their bacterial ancestry, mitochondrial DNA (mtDNA) is structurally similar to bacterial DNA.^12^ Once released from damaged mitochondria, mtDNA promotes the recruitment of various inflammatory cell types, leading to the production of inflammatory cytokines and the initiation of immune responses.^13^^,^^14^ Recently, increasing evidence has suggested that mtDNA contributes to the progression of various non-infectious diseases, including obesity and heart failure.^15^^,^^16^ We and others have observed the accumulation of impaired mitochondria in the hypertrophic heart following pressure overload,^17^^,^^18^ which may facilitate mtDNA release and STING activation, and ultimately, cardiac dysfunction. To protect against the damage caused by dysfunctional mitochondria, cells have developed quality control mechanisms to selectively sequester and degrade aberrant mitochondria, a biological process known as mitophagy.^19^ Mitophagy is primarily regulated by the cytosolic E3 ubiquitin ligase Parkin and PTEN-induced putative kinase-1 (PINK), which are located within the outer mitochondrial membrane.^20^ Although previous studies have indicated that insufficient mitophagy aggravates heart failure and inflammasome activation,^21^^,^^22^ whether mitophagy is involved in the activation of STING during cardiac remodeling is yet to be elucidated.

Given the important roles of STING and mitophagy in sterile inflammation and cardiac dysfunction, we hypothesized that impaired mitophagy aggravated cardiac remodeling by triggering STING activation. In the present study, the roles of STING and Parkin-mediated mitophagy were investigated in pressure overload-induced cardiac remodeling. We identified that genetically ablating STING expression protects the heart against pressure overload, and that impaired mitophagy is associated with increased cytosolic mtDNA accumulation and STING activation, contributing to cardiac remodeling. These findings suggest that mitophagic modulation, together with inhibiting STING activation, may be a potential therapeutic approach for cardiac remodeling.

## Materials and methods

### Reagents

Antibodies against the following targets were used in the present study: STING and IRF3 (Cell Signaling Technology, Inc., USA); α-SMA (α smooth muscle actin), p-IRF3, Parkin, PINK, and GAPDH (Affinity Biosciences, China); and vimentin, collagen 1 and collagen 3 (Wuhan Sanying Biotechnology, China). PMI and the selective diABZI STING receptor agonist-1 (CAS No.: 2138299-34-8) were obtained from MedChemExpress (USA). Fetal bovine serum was provided by Biological Industries (Israel), and siRNAs targeting STING and Parkin were designed and synthesized by Shanghai Qingke Biotechnology Co., Ltd. (China).

### Mice and treatments

Male C57BL/6 mice (8 weeks) were used in this study. All experimental procedures were approved by the Chongqing Medical University Committee on Animal Care according to the guidelines from Directive 2010/63/EU of the European Parliament on the protection of animals used for scientific purposes or the current NIH guidelines. The number of animals used in each experiment is indicated in the corresponding figures and accompanying legends.

The mouse model of pressure overload-induced cardiac remodeling was induced by transverse aortic constriction (TAC). Briefly, mice were anesthetized using isoflurane (4% for induction, 2% for maintenance) via inhalation, and the vessel between the innominate and carotid artery was ligated using a 27-gauge blunt needle. Sham-operated mice underwent the same procedure but without ligation. The surgery was performed by individuals blinded to the mouse treatment grouping.

To investigate the role of STING in pressure overload-induced cardiac remodeling, STING-Flox mice were obtained from Shanghai Biomodel Organism Science & Technology Development Co., Ltd. (NM-CKO-00014), and CAG-cre mice were obtained from The Jackson Laboratory (004682). *STING*^*fl/fl*^;*CAG*^*cre/+*^ were defined as STING knockout (STING KO) mice, and the corresponding *STING*^*fl/fl*^ mice were used as controls. To determine the role of Parkin-mediated mitophagy in STING activation, the mice were infected with lentivirus expressing Parkin siRNAs (Hanbio Biotechnology Co., Ltd., China) via intramyocardial injection, before TAC surgery. The expression of Parkin in the heart was measured at 1, 2, and 4 weeks post-infection and TAC surgery. At the end of the study, echocardiography was performed to assess cardiac function. The echocardiographic assessment was carried out 3 times, and the average value was taken.

### CF isolation, culture, transfection, and mtDNA depletion

CFs were isolated from 1-to-3-day-old neonatal C57BL/6 mice or TAC mice by enzymatic digestion. CFs were cultured in DMEM supplemented with 10% fetal bovine serum. For siRNA transfection, cells were cultured in serum-free DMEM for 12 h, and siRNA transfection was then conducted using Lipofectamine® RNAiMax (Invitrogen; Thermo Fisher Scientific, Inc., USA) according to the manufacturer's instructions. To deplete mtDNA in CFs, cells were cultured with ethidium bromide (EtBr) at 50, 100, or 200 ng/mL EtBr for 4 days, respectively.

### Histological analysis

Heart samples were collected from each group at the indicated time points after TAC. Subsequently, the hearts were perfused with cold PBS, fixed in 4% paraformaldehyde, and embedded in paraffin. Seven-μm-thickness cross-sections were de-paraffinized and rehydrated. For the evaluation of interstitial fibrosis, hematoxylin and eosin (H&E) staining and Masson's trichrome staining were performed using commercial kits (Beijing Solarbio Science & Technology Co., Ltd., China) according to the manufacturer's protocol.

Immunofluorescent staining was performed to evaluate CF phenotype. Briefly, CFs were fixed with 4% paraformaldehyde and blocked with 10% goat serum at room temperature for 30 min. Then, the cells were incubated with anti-α-SMA (1:300) antibodies at 4 °C overnight, followed by a FITC-conjugated secondary antibody at room temperature (in the dark) for 1 h. The cell nuclei were subjected to DAPI staining for 5 min, and subsequently observed under a fluorescence microscope (Leica Microsystems, Inc., USA).

Ultrastructure analysis was conducted using transmission electron microscopy. Briefly, heart samples were obtained from the left ventricle and then fixed in 2.5% glutaraldehyde (Beijing Solarbio Science & Technology Co., Ltd., China). The samples were viewed using a Hitachi H7500 TEM transmission electron microscope (Hitachi, Ltd., Japan). Images were captured at a magnification of 8000× for detecting mitochondrial size and number and at 20,000× magnification for detecting mitophagy.

Lyso-tracker and mito-tracker double staining was used for evaluating mitophagy in CFs. Briefly, CFs were incubated in DMEM supplemented with 70 nM Lyso-Tracker Red and 100 nM Mito-Tracker Green (Beyotime Institute of Biotechnology, China) at 37 °C for 25 min. The medium was replaced with fresh DMEM and images were captured using a Nikon A1 Plus laser scanning confocal microscopy microscope (Nikon, Inc., Japan).

### Detection of ATP and MDA levels and SOD activity

Cardiac ATP and malondialdehyde (MDA) levels and superoxide dismutase (SOD) activity were measured using ATP assay kits, lipid peroxidation MDA assay kits, and SOD assay kits (all Beyotime Institute of Biotechnology, China) respectively, according to the manufacturer's instructions.

### Western blotting

Total protein was extracted from heart tissues or CFs using a column tissue and cell protein extraction kit (EpiZyme, Inc., China). The protein concentration was measured with a BCA protein assay kit (Beyotime Institute of Biotechnology, China). Samples were then incubated with a loading buffer at 60 °C for 30 min. After SDS-PAGE, the samples were transferred to a PVDF membrane and probed with primary antibody at 4 °C overnight. The membranes were then incubated with the corresponding secondary antibodies at room temperature for 1–1.5 h. Images were obtained using ECL-plus reagent (Biosharp Life Sciences, China) and analyzed with Image J.

### Quantitative real-time PCR

Total RNA was isolated from heart tissues and CFs using TRIzol® reagent (Invitrogen; Thermo Fisher Scientific, Inc., USA), and 1 μg RNA was then reverse-transcribed using a Reverse Transcription Kit (Takara Bio, Inc., Japan). For cytosolic mtDNA detection, the DNA in the cytosol and total DNA were extracted as described previously.^16^ Real-time PCR was performed using SYBR Green (Takara Bio, Inc., Japan). The primers and sequences used in this study are displayed in Table S1.

### Statistical analysis

The data were presented as mean ± standard error of mean (SEM). Comparations between groups were performed using one- or two-way ANOVA. Corrections for multiple comparisons were performed using Tukey's or Sidak's test. *P* < 0.05 was considered statistically significant. All data were analyzed using GraphPad Prism 8 (GraphPad Software, Inc., USA).

## Results

### STING deficiency alleviates inflammation and pathogenetic cardiac remodeling in TAC mice

**Figure 1:**
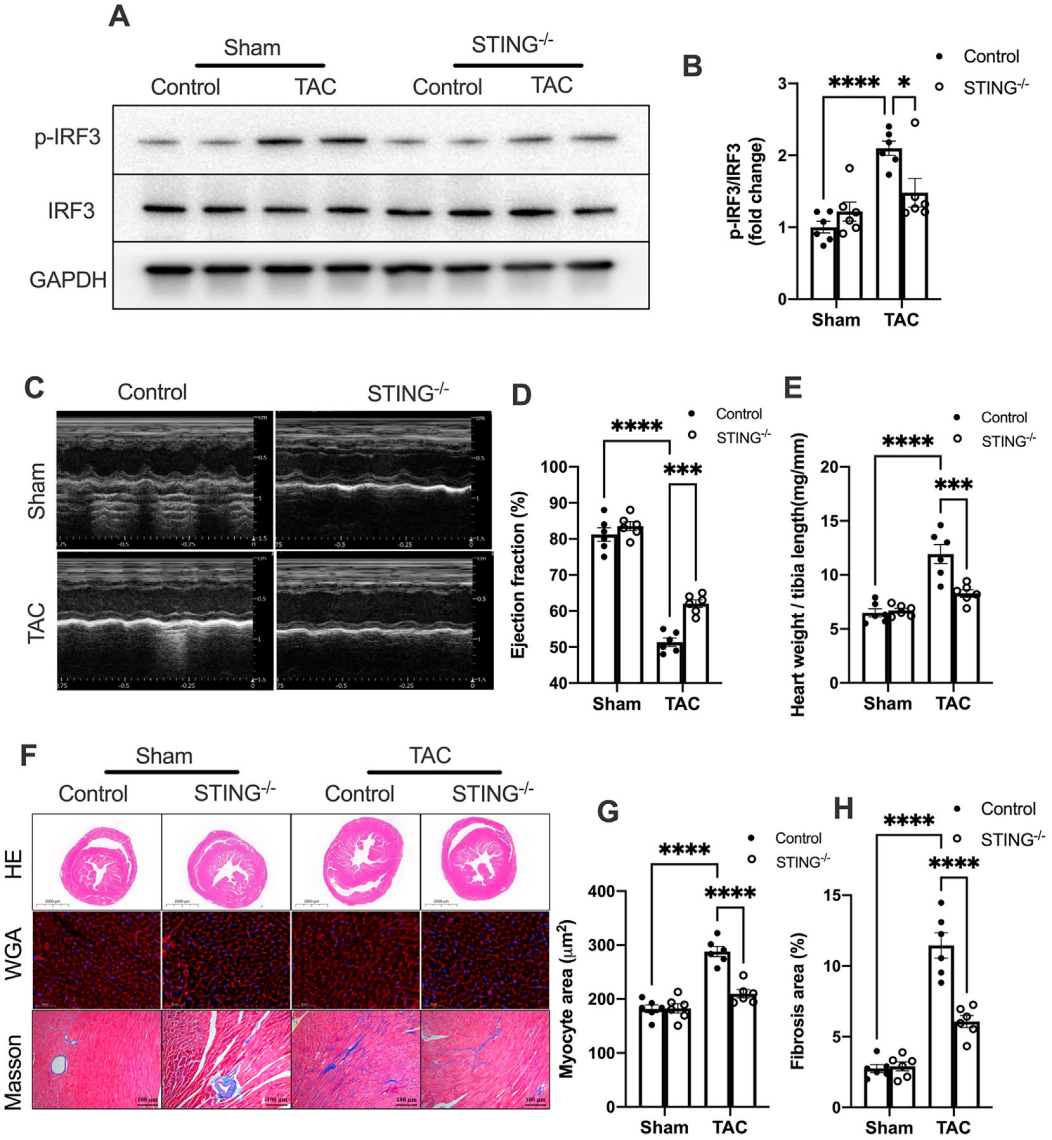
STING deficiency prevents pressure overload-induced cardiac remodeling. **(A)** STING deficiency attenuated STING pathway activation in TAC hearts. Quantitative results of **(B)** p-IRF3/IRF3 Western blot analysis (*n* = 6). **(C)** Representative M-mode images of echocardiography for each group. **(D)** Statistical analysis of cardiac function indexes LVEF (%). **(E)** The ratio of heart weight-to-tibia length (*n* = 6). **(F)** Representative H&E staining, wheat germ agglutinin (WGA) staining, and Masson's trichrome staining in the heart tissue. Scale bar, 100 μm. **(G, H)** Quantitative results of the myocyte and fibrotic area (*n* = 6). The data were presented as mean ± SEM; ∗*P* < 0.05, ∗∗∗*P* < 0.001, ∗∗∗∗*P* < 0.0001. STING, stimulator of interferon genes; TAC, transverse aortic constriction; IRF3, interferon regulatory factor 3; p-, phosphor-.

**Figure 2:**
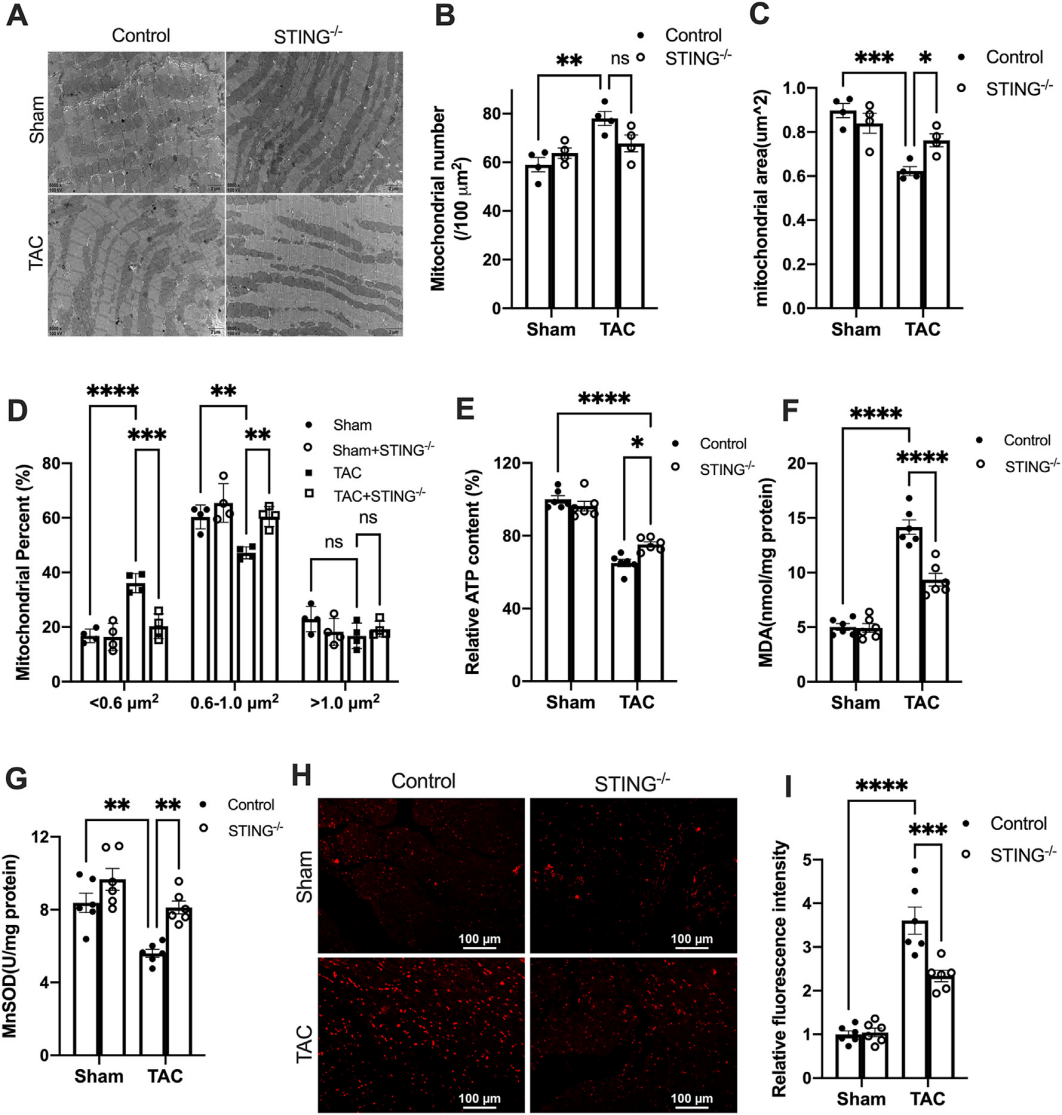
STING deficiency attenuates mitochondrial fission and oxidative stress. **(A)** Representative transmission electron microscopic images of heart cross-sections (scale bar = 2 μm). **(B)** Average mitochondrial number per μm^2^ (*n* = 4). **(C)** Mean mitochondrial size (*n* = 4). **(D)** Percentage of mitochondria in different size groups (*n* = 4). **(E)** STING deficiency increased ATP levels in the hearts of TAC mice. **(F)** MDA levels and **(G)** MnSOD activity in the hearts of control or STING KO mice 4 weeks after TAC surgery (*n* = 6). **(H, I)** Superoxide levels in cardiac tissue were detected using dihydroethidium. Representative images are shown in (H) and quantitative results are shown in (I) (*n* = 6). Scale bar, 100 μm. The data were presented as mean ± SEM. ∗*P* < 0.05, ∗∗*P* < 0.01, ∗∗∗*P* < 0.001, ∗∗∗∗*P* < 0.0001. STING, stimulator of interferon genes; TAC, transverse aortic constriction; KO, knockout; MnSOD, manganese superoxide dismutase; MDA, malondialdehyde; α-SMA, α smooth muscle actin.

We first investigated STING pathway activation in the hearts of TAC mice. Western blot analysis demonstrated a significant increase in STING expression and IRF3 phosphorylation at 3 weeks post-surgery (Fig. S1A–C). In addition, the mRNA expression levels of prototypical IRF3-dependent cytokines (IFNβ) and IFN-stimulated nuclear genes (Ifit1 and Ifit2) were also elevated (Fig. S1D–F), indicating that pressure overload activates the STING pathway in the hearts of TAC mice. To determine the role of STING in pressure overload-induced cardiac remodeling, STING KO mice were subjected to TAC surgery. Compared with the controls, STING deficiency almost completely ablated the increase in IRF3 phosphorylation in the hearts of TAC mice (Fig. 1A, B), as well as the elevated expression of IFNβ and ISGs (Fig. S1G–I). Consistently, STING deficiency significantly restored the cardiac pump function (Fig. 1C–E), and mitigated cardiac hypertrophy and interstitial fibrosis, as evidenced by reduced heart weight-to-tibia length ratio (Fig. 2E), cardiomyocyte cross-sectional area and fibrosis area (Fig. 2F–H). In summary, these findings demonstrate that STING contributes to pressure overload-induced cardiac remodeling and that targeting STING may be a potential therapeutic strategy for pressure overload-associated stress.

### STING deficiency attenuates mitochondrial fission and oxidative stress

mtDNA released from impaired mitochondria leads to STING activation, and STING also induces mitochondria dysfunction in turn.^23^^,^^24^ We and others have demonstrated that mitochondrial dysfunction induces oxidative stress and then contributes to cardiac remodeling and heart failure.^17^^,^^18^ Thus, we examined the potential impact of STING expression disruption on mitochondrial fragmentation and oxidative stress. Pressure overload resulted in mitochondrial fission, as evidenced by increased mitochondrial numbers (Fig. 2A, B) and decreased mitochondrial size (Fig. 2A, C) in the hearts of TAC mice. Furthermore, pressure overload significantly increased the percentage of mitochondria <0.6 μm^2^, but reduced the percentage of mitochondria between 0.6 μm^2^ and 1.0 μm^2^ (Fig. 2D). Pressure overload also decreased cardiac ATP level (Fig. 2E), providing further evidence of mitochondrial damage in the heart. Moreover, myocardial MDA levels (Fig. 2F) significantly increased, while SOD activity decreased (Fig. 2G) in the hearts of the TAC mice. The DHE staining also confirmed the increased myocardial superoxide anion production (Fig. 2H, I). Collectively, these findings suggest that pressure overload enhances oxidative stress in TAC hearts. In contrast, disrupting STING expression attenuated mitochondrial fragmentation and dysfunction, as well as excessive oxidative stress in the hearts of TAC mice (Fig. 2). However, STING deficiency has no significant effects on PINK and Parkin expression in TAC hearts (Fig. S2). These two lines of evidence indicate that suppressing STING may mitigate mitochondria fission and oxidative stress in the heart, which may be responsible for improved cardiac remodeling.

### STING exaggerates the differentiation and proliferation of CFs

**Figure 3:**
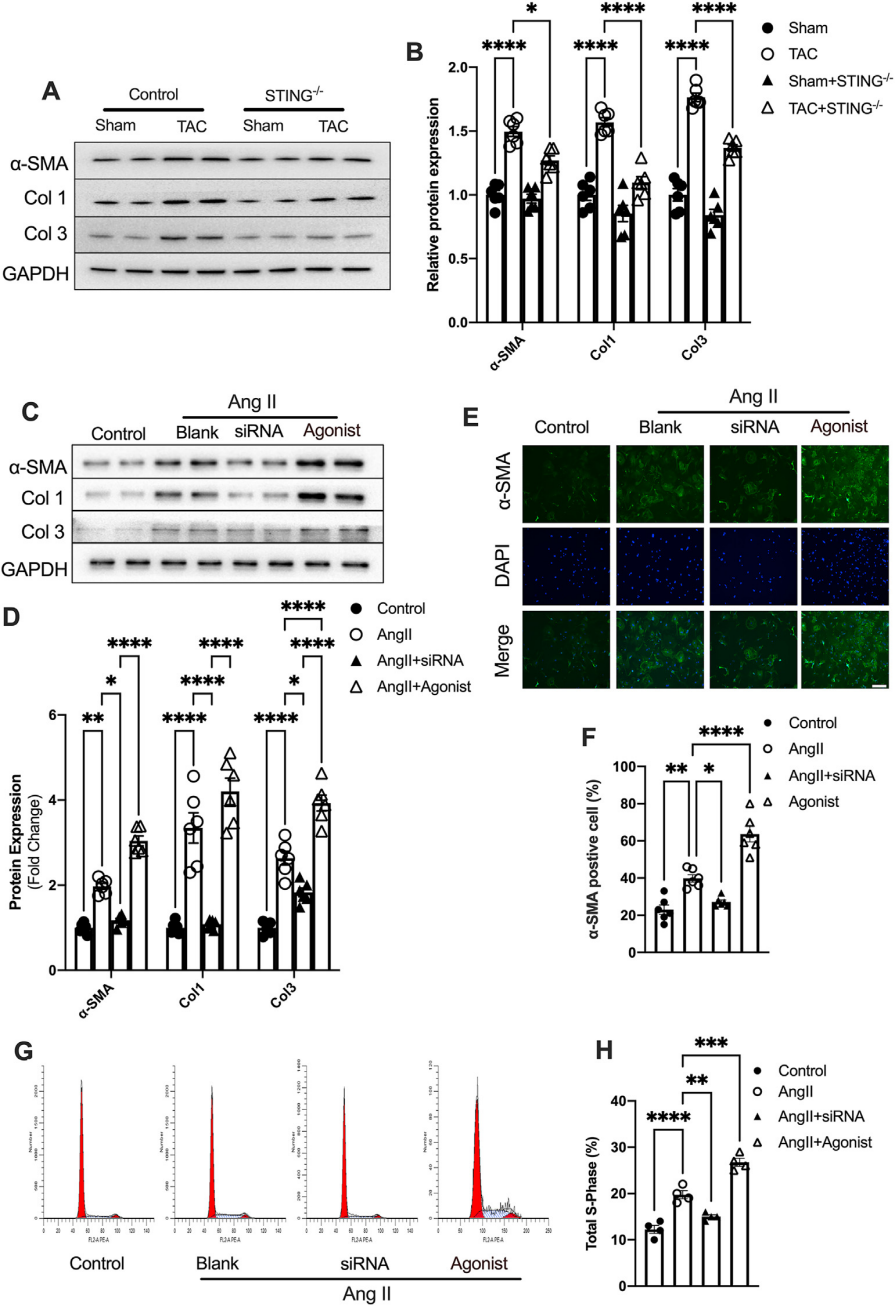
STING exaggerates Ang II-induced differentiation and proliferation of CFs. **(A)** Protein levels of α-SMA and collagen (type I and III) in isolated CFs from TAC hearts with or without STING knockout. The quantifications are shown in **(B)** (*n* = 6). **(C)** Protein levels of α-SMA and collagen (type I and III) in CFs exposed to Ang II for 24 h with siRNA-STING or a selective diABZI STING receptor agonist-1 (agonist). The quantifications are shown in **(D)** (*n* = 6). **(E)** Immunofluorescence staining of α-SMA in CFs. Scale bar, 200 μm. **(F)** α-SMA labeled cells (*n* = 6). **(G)** The cell cycle was assessed by flow cytometry. **(H)** The percentages of S-phase CFs after Ang II treatment (*n* = 6). The data were presented as mean ± SEM. ∗*P* < 0.05, ∗∗*P* < 0.01, ∗∗∗*P* < 0.001, ∗∗∗∗*P* < 0.0001. STING, stimulator of interferon genes; Ang II, angiotensin II; CFs, cardiac fibroblasts; IRF3, interferon regulatory factor 3; p-, phospho; α-SMA, α smooth muscle actin.

CFs are not only the main orchestrators of interstitial fibrosis but also exhibit functions in inflammation and immune response.^25^ To determine whether STING is directly involved in fibroblast activation, CFs were isolated from TAC hearts. The results showed that STING deficiency significantly blunted the increased expression of α-SMA and collagen I and III (Fig. 3A, B). Moreover, in isolated CFs from neonatal mice, Ang II stimulation increased STING expression and IRF3 phosphorylation (Fig. S3A, B). Concurrent with STING activation, the expression of α-SMA and collagen I and III were also increased (Fig. S3A, B). In order to gain insights into the effects of STING on fibrotic cell activation, we next suppressed or enhanced the activation of the STING pathway using small interfering (si) RNAs or the selective STING receptor agonist, respectively. The effects of siRNA-mediated STING knockdown and agonist-induced STING activation were confirmed by Western blotting (Fig. S4). As predicted, STING knockdown inhibited the Ang II-induced increase in α-SMA and collagen I and III expression in CFs, while the STING agonist further enhanced the expression of collagen and α-SMA, as evidenced by Western blotting and immunofluorescence staining (Fig. 3C–F). The proliferation of CFs is another feature of cardiac fibrosis. Flow cytometry analysis indicated that STING knockdown significantly suppressed fibroblast proliferation. Conversely, the STING agonist promoted the Ang II-induced proliferation of CFs (Fig. 3G, H). Taken together, those data confirm that STING promotes fibroblast proliferation and the conversion of fibroblasts into myofibroblasts, which exaggerates interstitial fibrosis.

### Mitophagy deficiency facilitates STING pathway activation

**Figure 4:**
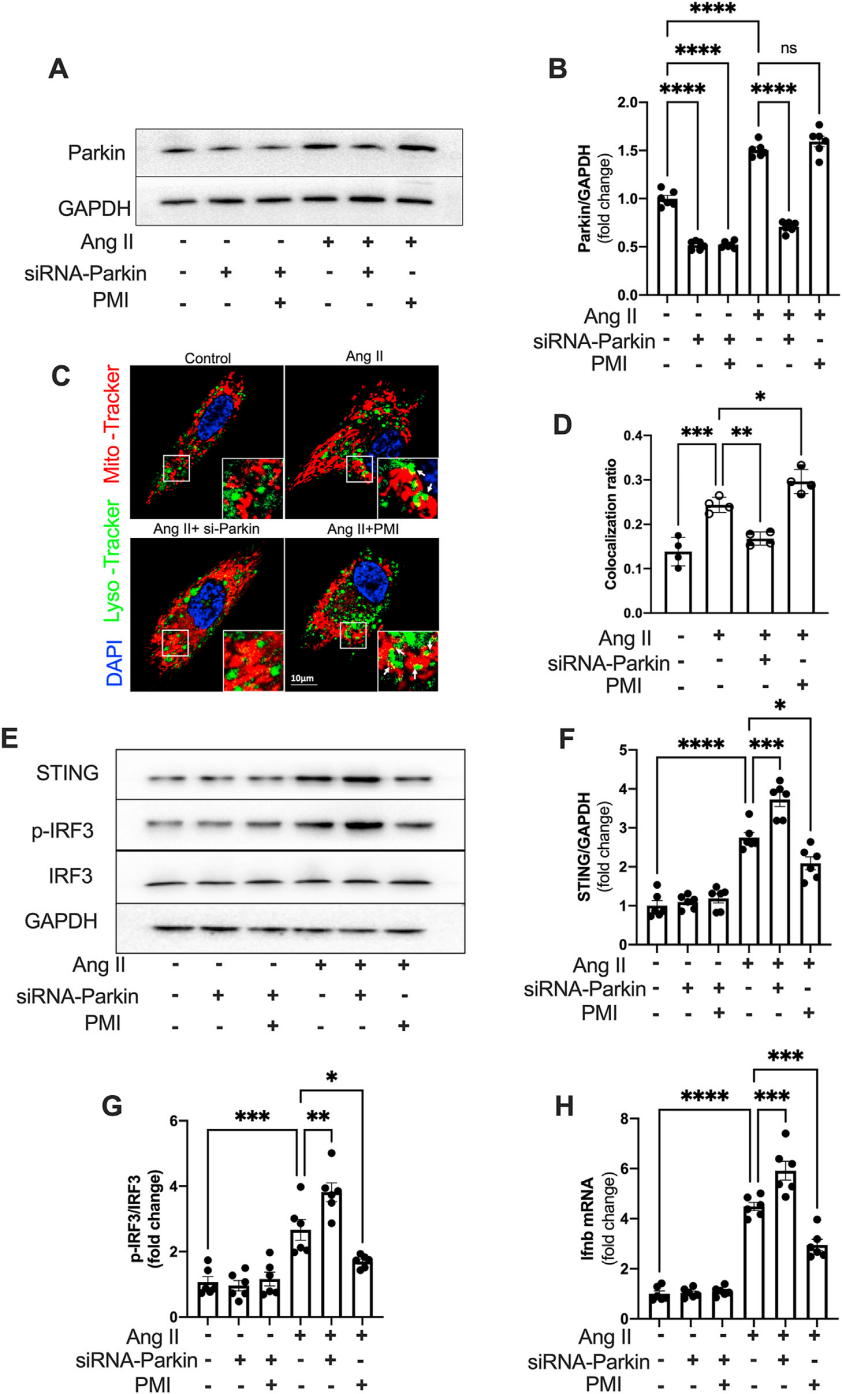
Mitophagy deficiency facilitates STING pathway activation. **(A)** Protein levels of Parkin in CFs exposed to Ang II (100 nM) for 24 h with siRNA-Parkin or PMI. Quantifications are shown in **(B)** (*n* = 6). **(C)** CF mitophagy was confirmed by the co-location of mito-tracker green and lyso-tracker red after exposure to Ang II for 24 h with siRNA-Parkin or PMI. Scale bar, 10 μm. The colocalization ratios are shown in **(D)** (*n* = 4). **(E**–**G)** Protein levels and quantification of STING, p-IRF3, and IRF3 in CFs exposed to Ang II with siRNA-Parkin or PMI (*n* = 6). **(H)** The mRNA expression of IFNβ was assessed by qPCR (*n* = 6). The data were presented as mean ± SEM. ∗*P* < 0.05, ∗∗*P* < 0.01, ∗∗∗*P* < 0.001, ∗∗∗∗*P* < 0.0001. STING, stimulator of interferon genes; ns, not significant; CFs, cardiac fibroblasts; siRNA, siRNA-STING; TAC, transverse aortic constriction; PMI, P62-mediated mitophagy inducer; Ang II, angiotensin II; IRF3, interferon regulatory factor 3; p-, phospho.

**Figure 5:**
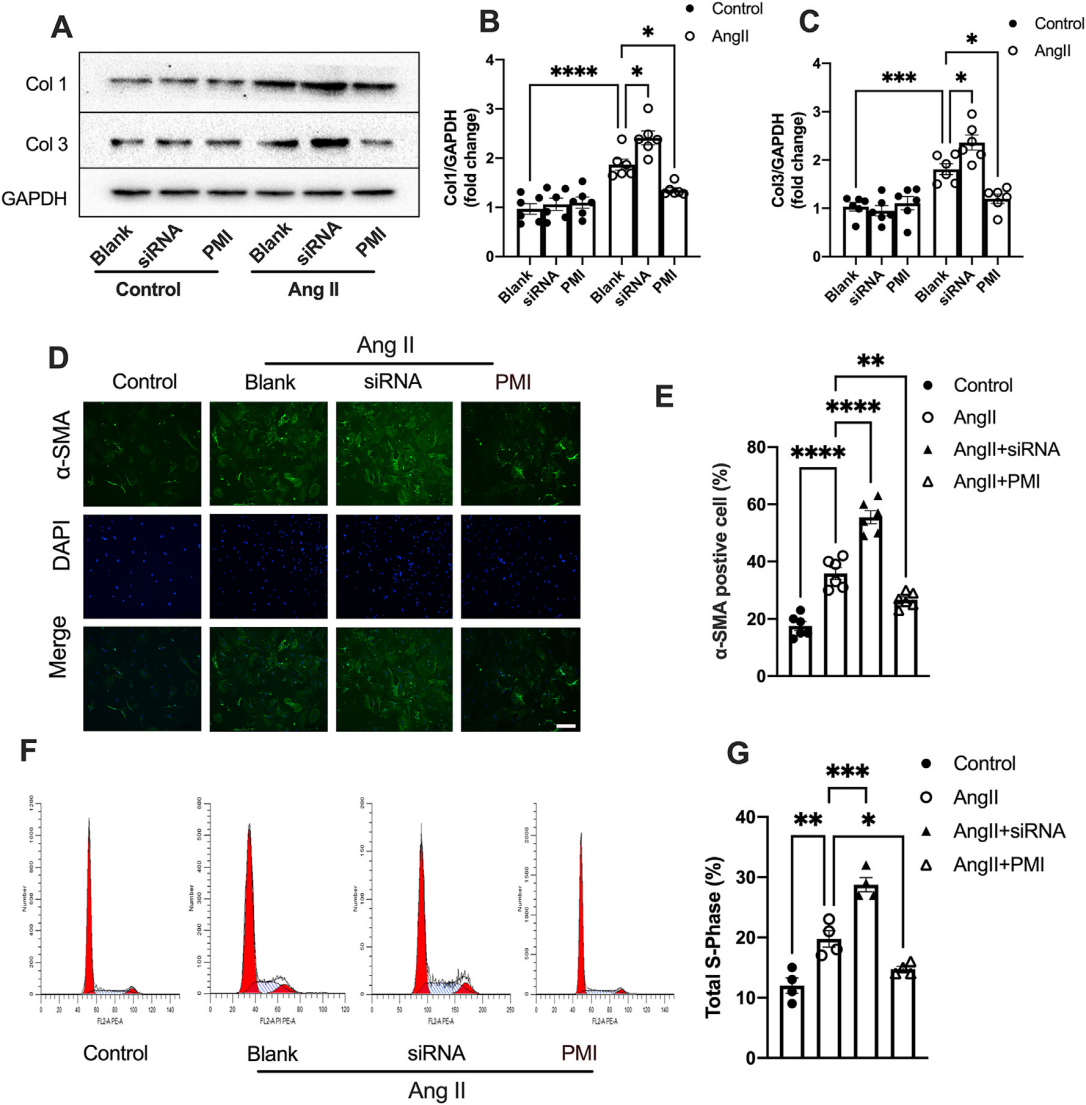
Mitophagy deficiency promotes Ang II-induced phenotypic switching of CFs. **(A)** Protein levels of collagen I and III were measured in CFs. The quantifications are shown in **(B, C)** (*n* = 6). **(D, E)** Representative immunofluorescence staining and quantitative analysis of α-SMA in CFs exposed to Ang II with siRNA-Parkin or PMI (*n* = 6). Scale bar, 200 μm. **(F)** The cell cycle was assessed by flow cytometry. **(G)** The percentages of S-phase CFs after Ang II treatment (*n* = 6). The data were presented as mean ± SEM. ∗*P* < 0.05, ∗∗*P* < 0.01, ∗∗∗*P* < 0.001, ∗∗∗∗*P* < 0.0001. Ang II, angiotensin II; CFs, cardiac fibroblasts; siRNA, siRNA-Parkin; PMI, P62-mediated mitophagy inducer; α-SMA, α smooth muscle actin; Col, collagen.

Given the important role of mitophagy in mitochondrial quality control and STING pathway activation,^26^^,^^27^ we next aimed to investigate the association between mitophagy and STING activation in the TAC heart. Pressure overload increased PINK/Parkin expression at one week following TAC, which was maintained for up to four weeks (Fig. S5A, B). However, the expression of FUNDC1 and BNIP were not significantly changed in TAC hearts (Fig. S5A, B), indicating it is Parkin-dependent mitophagy that is mainly involved in TAC-induced pathological cardiac remodeling. Similarly, Ang II elevated the expression of PINK and Parkin in CFs (Fig. S5C–E). To confirm that mitophagy regulates STING activation, we inhibited mitophagy by suppressing Parkin expression with siRNAs or activated mitophagy using a P62-mediated mitophagy inducer (PMI). The successful knockdown of Parkin was confirmed by Western blotting (Fig. S6; Fig. 4A, B). Co-localization of mito-tracker and lyso-tracker is considered a marker of mitophagy, and the results confirmed that Ang II activated mitophagy in CFs, which was retarded by Parkin knockdown and enhanced by PMI (Fig. 4C, D). In addition, Parkin knockdown promoted Ang II-induced STING activation (Fig. 4E and F), which is evidenced by increased IRF3 phosphorylation (Fig. 5G) and IFNβ expression (Fig. 5H). However, activating mitophagy almost entirely inhibited the activation of the STING pathway in Ang II-stimulated CFs (Fig. 5E–H). These data demonstrate the regulatory role of mitophagy in STING pathway activation.

### Mitophagy deficiency aggravates Ang II-induced phenotypic switching of CFs

We next investigated the role of mitophagy in the Ang II-induced differentiation and proliferation of CFs. Inhibiting mitophagy by knocking down Parkin further increased the expression of collagen and α-SMA in Ang II-stimulated CFs (Fig. 5A–E). In addition, the proliferation of CFs was also promoted by inhibiting mitophagy (Fig. 5F, G). However, increasing mitophagy mitigated the Ang II-induced differentiation and proliferation of CFs, as evidenced by decreased expression of collagen and α-SMA, as well as cell cycle arrest (Fig. 5A–G). In summary, those results confirm that mitophagy deficiency contributes to the adverse phenotypic switching of CFs.

### Blocking mitophagy contributes to cytosolic mtDNA accumulation and STING activation

**Figure 6:**
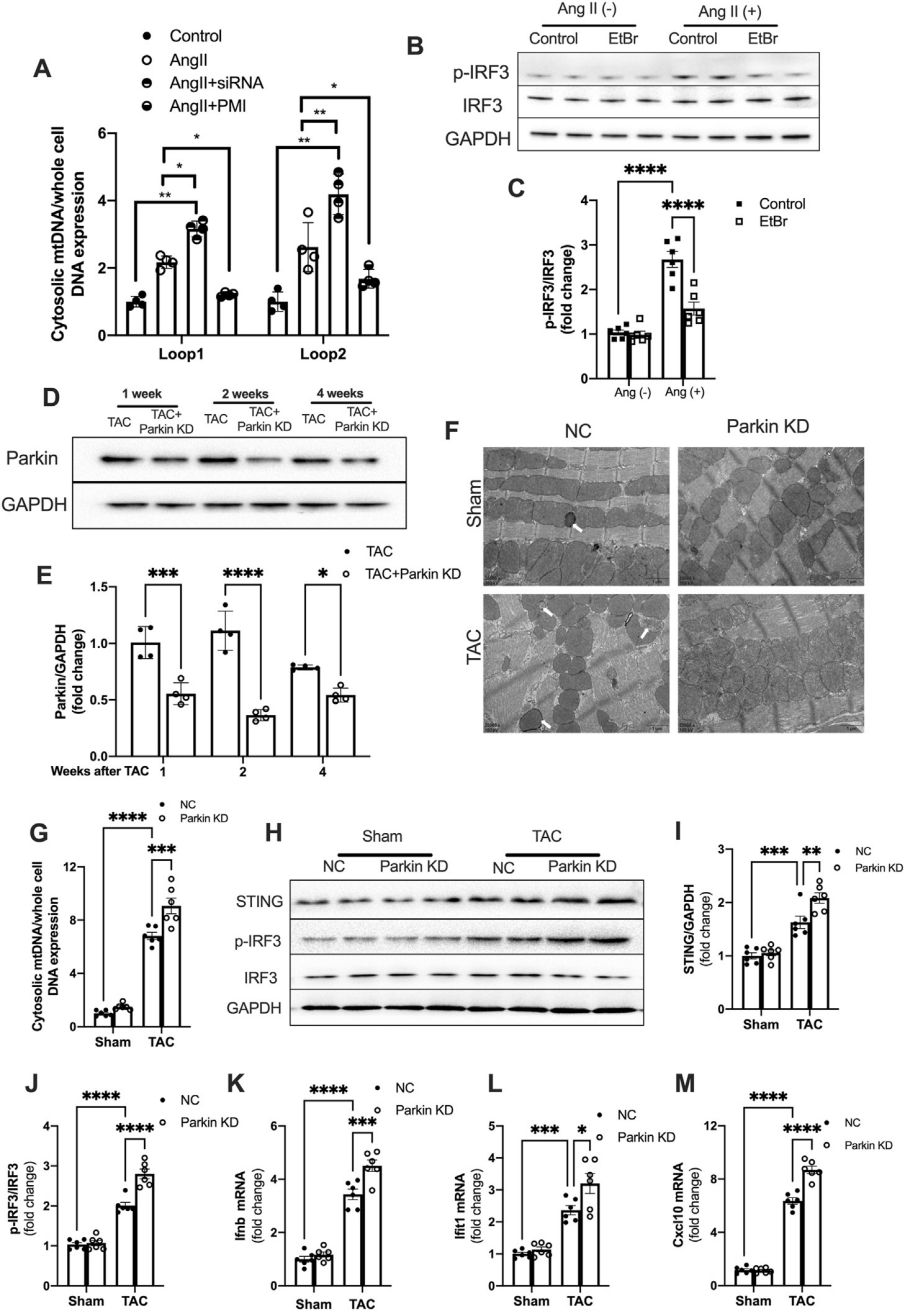
Blocking mitophagy contributes to cytosolic mtDNA accumulation and STING activation. **(A)** CF cytosolic mtDNA levels were measured with RT-PCR after exposure to Ang II for 24 h with siRNA-Parkin or PMI (*n* = 4). **(B, C)** mtDNA depletion blunted the effect of Ang II on activating STING-induced IRF3 phosphorylation (*n* = 6). **(D)** Parkin knockdown attenuated increased Parkin expression in the hearts of TAC mice. **(E)** Quantification of Parkin expression in the TAC heart (*n* = 4). **(F)** Transmission electron microscopy was used to detect mitophagy in the hearts of TAC mice. The white arrows represent mitophagy. Scale bar, 1 μm. **(G)** Cytosolic mtDNA levels in the heart were measured with RT-PCR at 4 weeks post-TAC (*n* = 6). **(H)** Parkin knockdown aggravated STING pathway activation in TAC hearts. **(I, J)** Qualification of STING and p-IRF3/IRF3 (*n* = 6). **(K, M)** Quantitative real-time PCR was performed to evaluate the transcript abundance of IFN-β and interferon-stimulated genes activated by the STING pathway (*n* = 6). The data were presented as mean ± SEM. ∗*P* < 0.05, ∗∗*P* < 0.01, ∗∗∗*P* < 0.001, ∗∗∗∗*P* < 0.0001. STING, stimulator of interferon genes; TAC, transverse aortic constriction; AV, adenovirus. EtBr, ethidium bromide; KD, knockdown; IRF3, interferon regulatory factor 3; p-, phospho.

Since TAC induces mitochondrial damage and mtDNA activates the STING pathway, we next investigated whether mitophagy was involved in the cytosolic mtDNA release thus leading to STING activation. Ang II increased cytosolic mtDNA accumulation in CFs (Fig. 6A). Inhibiting mitophagy by knocking down Parkin further increased the cytosolic mtDNA levels (Fig. 6A). In contrast, increasing mitophagy by PMI mitigated the Ang II-induced cytosolic mtDNA accumulation (Fig. 6A). To further explore the role of mtDNA in STING activation, we depleted mtDNA content in CFs using EtBr. The efficiency of mtDNA depletion was measured with RT-PCR (Fig. S7), and 200 ng/mL EtBr was used for the next experiments. Our results showed that Ang II-induced IRF3 phosphorylation was significantly blunted by mtDNA depletion (Fig. 6B, C). Those results indicate that mitophagy may regulate STING activation via cytosolic mtDNA accumulation.

To further clarify the role of mitophagy in cytosolic mtDNA accumulation and STING activation *in vivo*, Parkin expression was silenced and the efficiency of Parkin silencing was measured with Western blot assays at 1, 2, and 4 weeks after TAC surgery. The results demonstrate that Parkin expression was reduced by 31% at 1 week, 64% at 2 weeks, and 44% at 4 weeks post-TAC in the heart (Fig. 6D, E). Moreover, mitophagy was markedly reduced in the heart by silencing Parkin expression, as evidenced by the decreased number of autophagosomes enclosing the mitochondria (white arrows) with transmission electron microscopy (Fig. 6F). The cytosolic mtDNA level significantly increased in the cytosolic fraction of myocardium from TAC mice, and that was further elevated by inhibiting Parkin-mediated mitophagy (Fig. 6G). Moreover, increased STING expression and IRF3 phosphorylation were further elevated by inhibiting Parkin-mediated mitophagy in the hearts of TAC mice (Fig. 6H–J), as well as the increase in the transcription of IFN-β and ISGs activated by the STING pathway (Fig. 6K–M).

### Blocking mitophagy promotes pathogenetic cardiac remodeling in TAC mice

**Figure 7:**
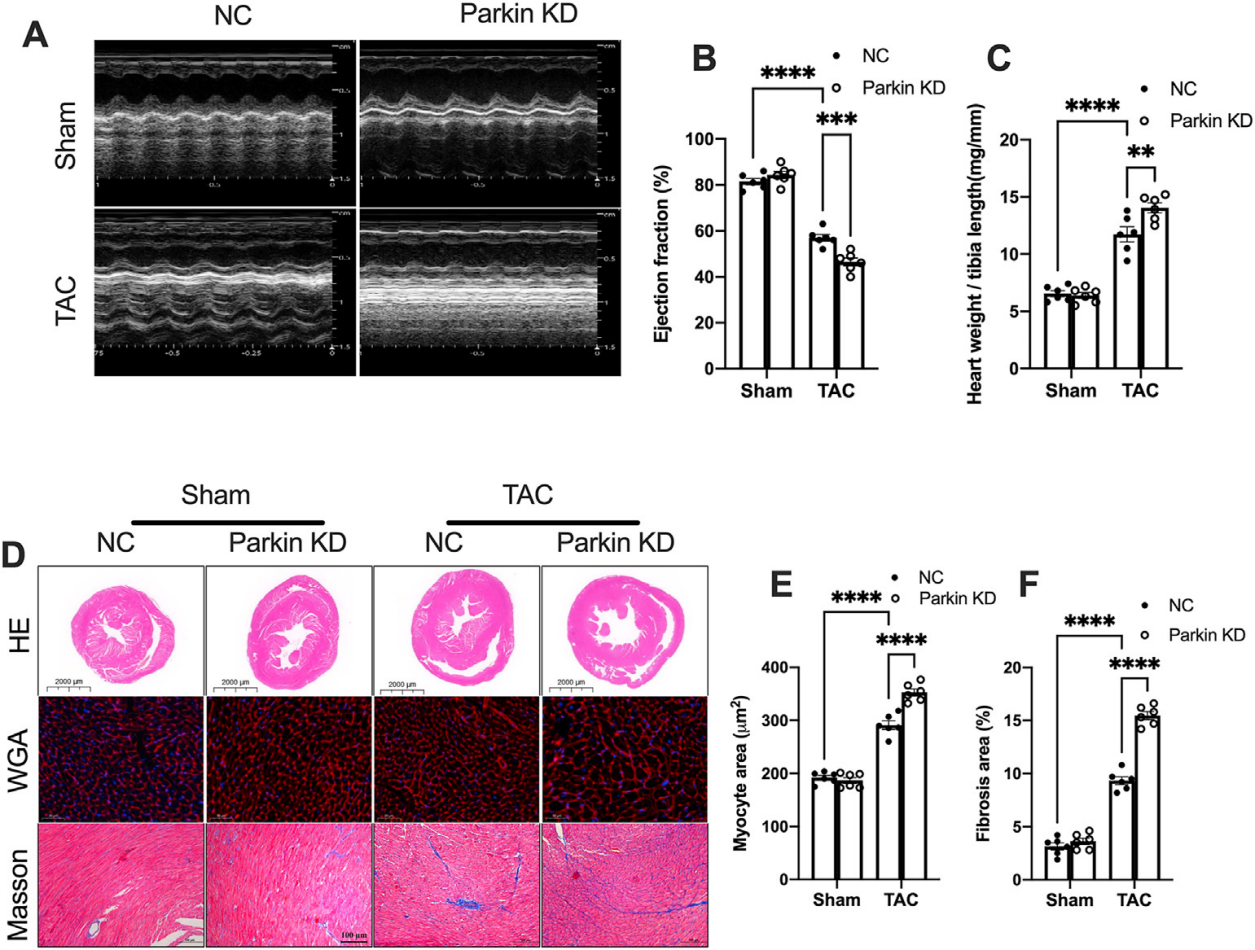
Blocking mitophagy promotes cardiac remodeling in TAC mice. **(A)** Representative M-mode images of echocardiography for each group. **(B)** Statistical analysis of cardiac function index LVEF (%). **(C)** The ratio of heart weight-to-tibia length (*n* = 6). **(D)** Representative H&E staining, wheat germ agglutinin (WGA) staining, and Masson's trichrome staining in the heart tissue. Scale bar, 100 μm. **(E, F)** Quantification of the myocyte and fibrotic area (*n* = 6). The data were presented as mean ± SEM. ∗∗*P* < 0.01, ∗∗∗*P* < 0.001, ∗∗∗∗*P* < 0.0001. TAC, transverse aortic constriction; AV, adenovirus; KD, knockdown; α-SMA, α smooth muscle actin.

As mitophagy deficiency increased cytosolic mtDNA accumulation and STING-induced inflammation, we thus evaluated the effects of mitophagy deficiency on cardiac remodeling in TAC mice. It was observed that Parkin knockdown decreased cardiac pump function (Fig. 7A, B), and however, increased the heart weight-to-tibia length ratio (Fig. 7C), cardiomyocyte cross-sectional area, and area of interstitial fibrosis (Fig. 7D–F) in the TAC heart. Collectively, these results suggest that inhibiting Parkin-mediated mitophagy promotes STING activation, and thus aggravates pressure overload-induced cardiac remodeling.

## Discussion

Pathological cardiac remodeling induced by pressure overload impairs ventricular compliance and leads to heart failure. Thus, research focusing on the molecular mechanisms underlying cardiac remodeling may highlight potential therapeutic options for those with heart failure. The results of the present study indicate that STING contributes to cardiac fibrosis and promotes adverse remodeling and that targeting STING protects the heart against pressure overload. In addition, mitophagy plays crucial roles in regulating STING activation. Mitophagy deficiency in the heart increased cytosolic mtDNA accumulation facilitating STING-mediated inflammation and aggravating cardiac remodeling in pressure overload-stressed hearts.

Cardiac fibrosis is a primary pathological feature during adverse cardiac remodeling. Cardiac fibrosis is characterized by the excessive proliferation of CFs and phenotypic switching of fibroblasts to myofibroblasts, which express more α-SMA and produce increased extracellular matrix proteins.^28^, ^29^, ^30^ In addition to their role in matrix remodeling, fibroblasts have been found to serve as sentinel cells that initiate the inflammatory response following myocardial injury.^31^^,^^32^ However, the signaling pathway initiating these inflammatory reactions in fibroblasts has not been well studied. STING is a newly discovered proinflammatory molecule that plays an important role in triggering sterile inflammation in non-infectious diseases.^33^^,^^34^ In the current study, we revealed that the STING pathway is activated in pressure overload-induced hearts, as well as Ang II-stimulated CFs. Suppressing the activation of STING-mediated inflammatory pathways prevented cardiac fibrosis and remodeling in the hearts of TAC mice. Furthermore, we directly confirmed that STING knockdown retarded Ang II-induced proliferation and phenotype switching to myofibroblasts in isolated CFs. These results suggest that STING is a critical molecule contributing to inflammation and cardiac fibrosis in pressure-overloaded hearts.

In accordance with our results, some other studies showed that STING expression contributed to pathological cardiac remodeling induced by pressure overload.^35^^,^^36^ Zhang et al reported that STING induced ER stress and aggravated cardiac remodeling.^36^ Hu et al also showed that increased cGAS-STING pathway activation was associated with apoptosis and oxidative, where its inhibition alleviated cardiac remodeling and improved cardiac function.^35^ However, Xiong et al proved that increased STING expression protected against cardiac dysfunction with cardiomyocyte-specific overexpressed STING transgenic mice.^37^ There exist several factors that could potentially account for the controversy. Firstly, STING plays multiple roles besides inducing inflammation, such as regulating autophagy and metabolism. Inhibiting STING expression in stressed hearts may mitigate cardiac dysfunction by alleviating inflammation and oxidative stress. However, it is worth noting that an augmented expression of STING could potentially yield benefits that surpass the injury via other cell biological functions as evidenced by Xiong et al. In addition, we and Hu's team employed TAC to induce cardiac remodeling. However, Zhang et al and Xiong et al utilized aortic banding for four weeks or six weeks to induce cardiac remodeling. Those differences in model construction may also lead to inconsistencies in the conclusion.

It has been established that excessive oxidative stress promotes collagen synthesis in fibroblasts, as well as the progression of remodeling.^38^^,^^39^ In addition, impaired mitochondria in the stressed heart are the major source of reactive oxygen species.^40^ Our findings also revealed that STING deficiency attenuates pressure overload-induced mitochondrial fission and improves mitochondrial function in the heart. Moreover, this increase in oxidative stress was mitigated by silencing STING expression. These findings suggest that STING deficiency improves mitochondrial dynamics and decreases oxidative stress levels in stressed hearts. However, further studies are required to confirm whether this is a direct effect of STING deficiency or secondary to the suppression of inflammation.

In addition to ATP production, mitochondria also contribute to the activation of immune responses by generating and releasing multiple DAMPs.^12^ Among them, DNA is released from impaired mitochondria into the cytosol and directly activates STING-mediated sterile inflammation.^41^^,^^42^ Mitophagy is a process that selectively clears aberrant mitochondria before they can cause damage, which also decreases the cytoplasmic accumulation of mitochondrial DNA released by impaired mitochondria.^43^^,^^44^ A previous study reported that inhibiting mitophagy exerts a strong inflammatory phenotype and increases cytosolic mtDNA levels in mice subject to exhaustive exercise, and loss of STING rescued this inflammation,^45^ suggesting that mitophagy may play roles in STING activation-induced inflammation. Although the role of mitophagy in regulating cardiac mitochondrial function and oxidative stress has been thoroughly investigated,^26^ whether mitophagy regulates the cytosolic mtDNA accumulation and STING-mediated inflammatory pathway as well as fibrosis in the stressed heart has not been studied. In this study, we suppressed mitophagy by silencing cardiac Parkin expression and observed greater cytosolic mtDNA accumulation and STING activation. Those findings confirmed the important role of mitophagy in regulating STING-induced inflammation via controlling cytosolic mtDNA accumulation. Moreover, our results demonstrate that mitophagy deficiency promotes interstitial fibrosis and pathological remodeling in pressure overload-stressed hearts, suggesting that mitophagy may be an upstream mechanism regulating STING-induced inflammation and cardiac injury.

One limitation of the current study is the absence of dependable tools for targeting myofibroblast-specific genes, resulting in the inability to selectively suppress the expression of STING and Parkin in fibroblasts. Thus, we cannot exclude the effects of other cells, such as cardiomyocytes, endotheliocytes, and macrophages, in the activation of STING. However, our *in vitro* experiments suggested a crucial role for mitophagy and the STING pathway in activating CFs and promoting fibrosis, highlighting the sentinel role of fibroblasts.

**Figure 8:**
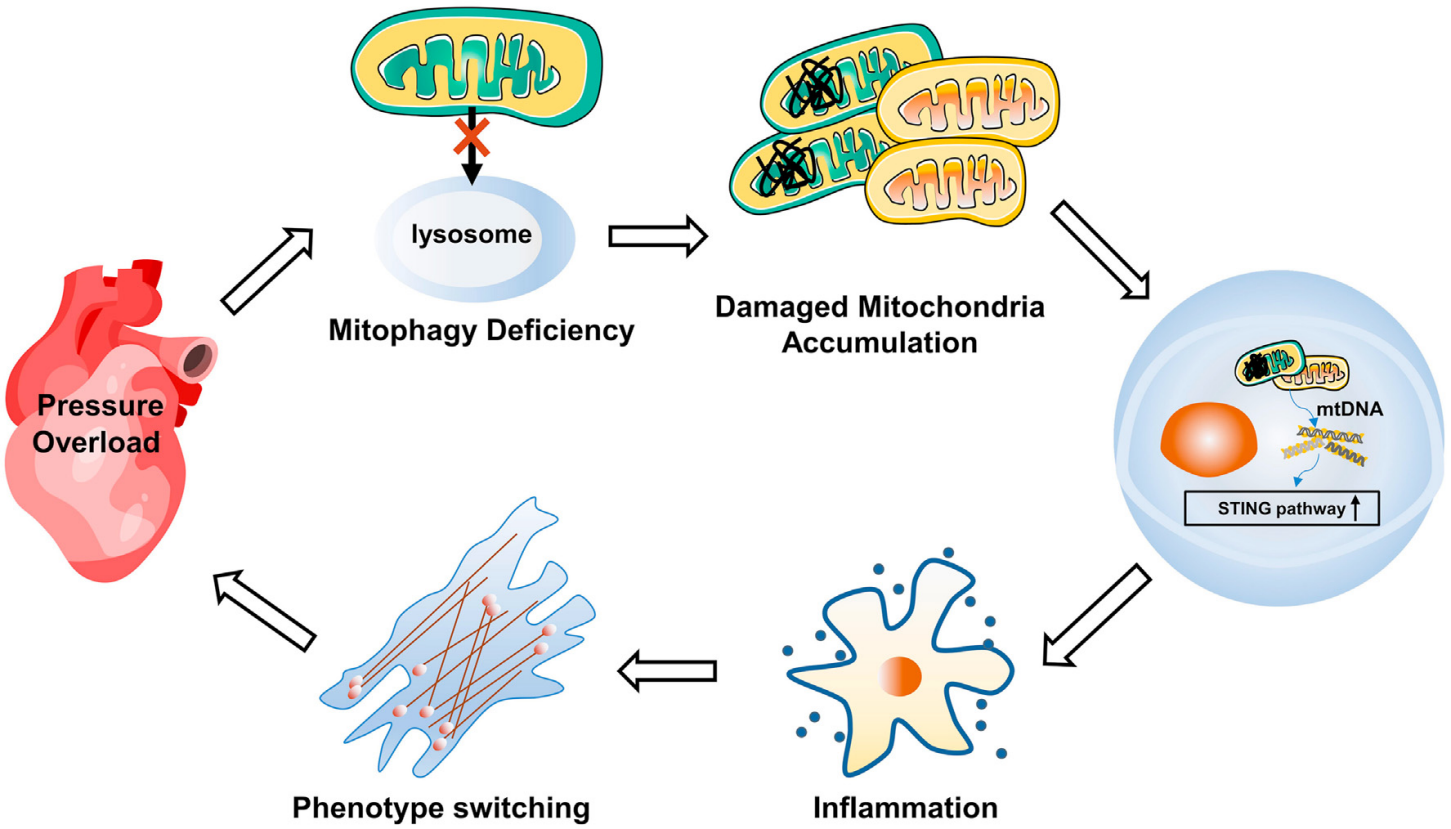
The schematic diagram illustrating that the impaired mitophagy and mtDNA accumulation leads to STING activation and thus contributes to cardiac remodeling in response to pressure overload.

In summary, our findings highlight the concept that mitophagy is involved in the regulation of inflammatory reactions (Fig. 8). In addition, we have demonstrated STING promotes pathogenetic cardiac remodeling by increasing fibroblast proliferation and myofibroblast formation in pressure-overload hearts. Targeting STING protects the heart against pressure overload, potentially by attenuating mitochondrial dysfunction and oxidative stress. Therefore, STING is suggested to be a potential therapeutic target for the prevention of cardiac remodeling induced by pressure overload.

## Conflict of interests

The authors declare no competing interests.

## Funding

This work was supported by grants from the 10.13039/501100001809Natural Science Foundation of China (No. 82070238), the 10.13039/501100002858China Postdoctoral Science Foundation (No. 2022M720601), the 10.13039/501100001809Natural Science Foundation of Chongqing, China (No. CSTB2022NSCQ-MSX0913), the Program for Youth Innovation in Future Medicine, Chongqing Medical University (No. W0168), the Science Fund of the First Affiliated Hospital of Chongqing Medical University (No. PYJJ2021-05), and the Postdoctoral Incubation Project of The First Affiliated Hospital of Chongqing Medical University (No. CYYY-BSHPYXM-202204).

## Data availability

All data needed to evaluate the conclusions in the paper are present in the paper and/or the Supplementary Materials. Additional data related to this paper may be requested from the authors.
