# Supplementary material for: Mitophagy deficiency activates stimulator of interferon genes activation and aggravates pathogenetic cardiac remodeling
Source: Genes Dis. 2023 Sep 2;11(6):101074. doi: 10.1016/j.gendis.2023.08.003 (PMC11399633; doi:10.1016/j.gendis.2023.08.003)
Supplement: Multimedia component 1 [file mmc1.docx]

**Supplement materials**

**
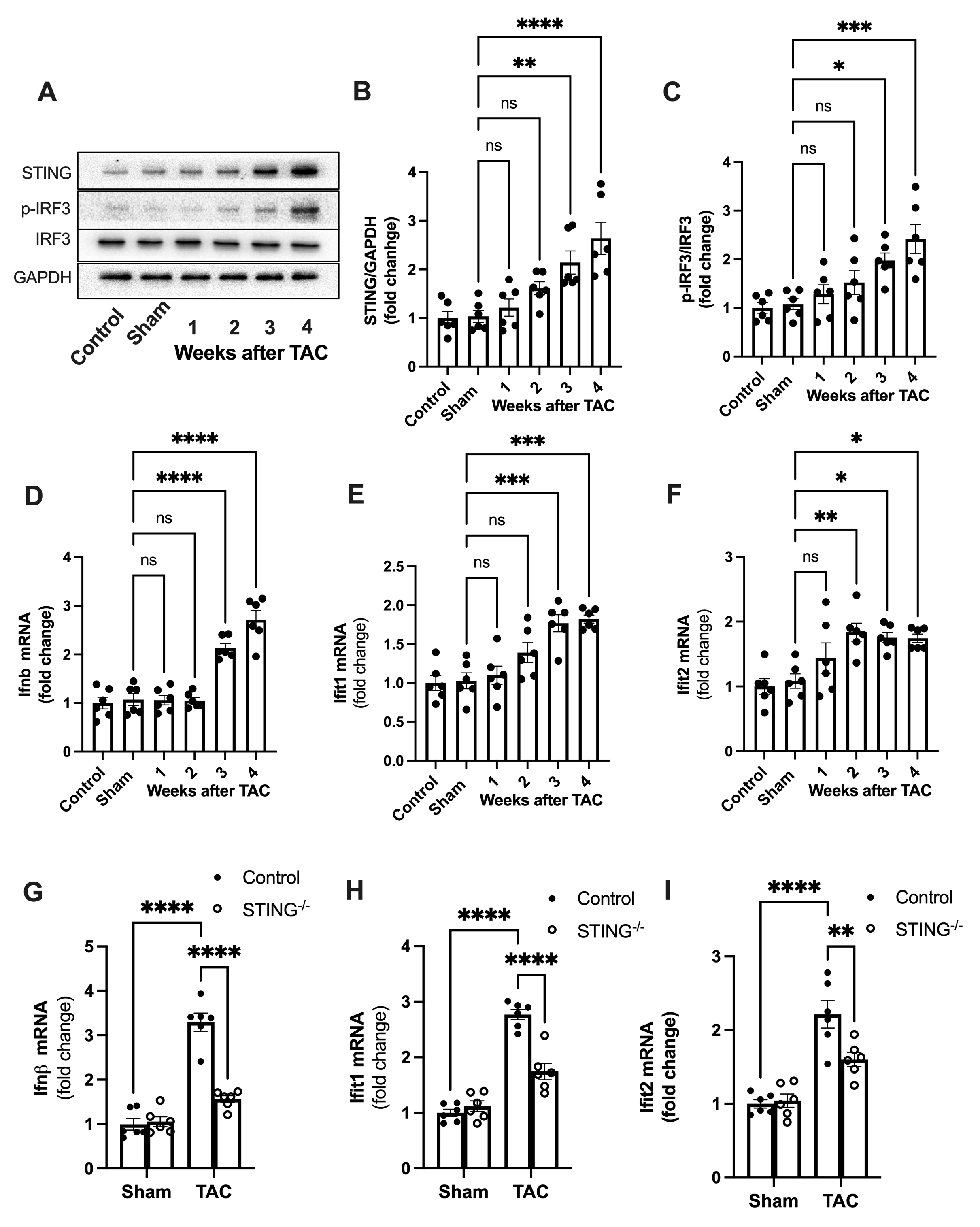
**

**Fig. S1** Pressure overload activates STING-induced inflammation in the heart of TAC mice. **(A)** Pressure overload increased STING expression and the phosphorylation of IRF3. Quantitative results of **(B)** STING and **(C)** p-IRF3/IRF3 western blot analysis (n=6). **(D–F)** Quantitative RT-PCR was performed to evaluate transcript abundances of IFN-β and interferon-stimulated genes (ISG) activated by STING pathway (n=6). **(G–I)** STING knockout reduced the transcript abundances of IFN-β and interferon-stimulated genes (ISG) activated by STING pathway in TAC hearts (n=6). Data are presented as the mean ± SEM; **P*<0.05, ***P*<0.01, ****P*<0.001 and *****P*<0.0001. ns, no significant. STING, stimulator of interferon genes. TAC, transverse aortic constriction. IRF3, interferon regulatory factor 3. p-, phosphor-.


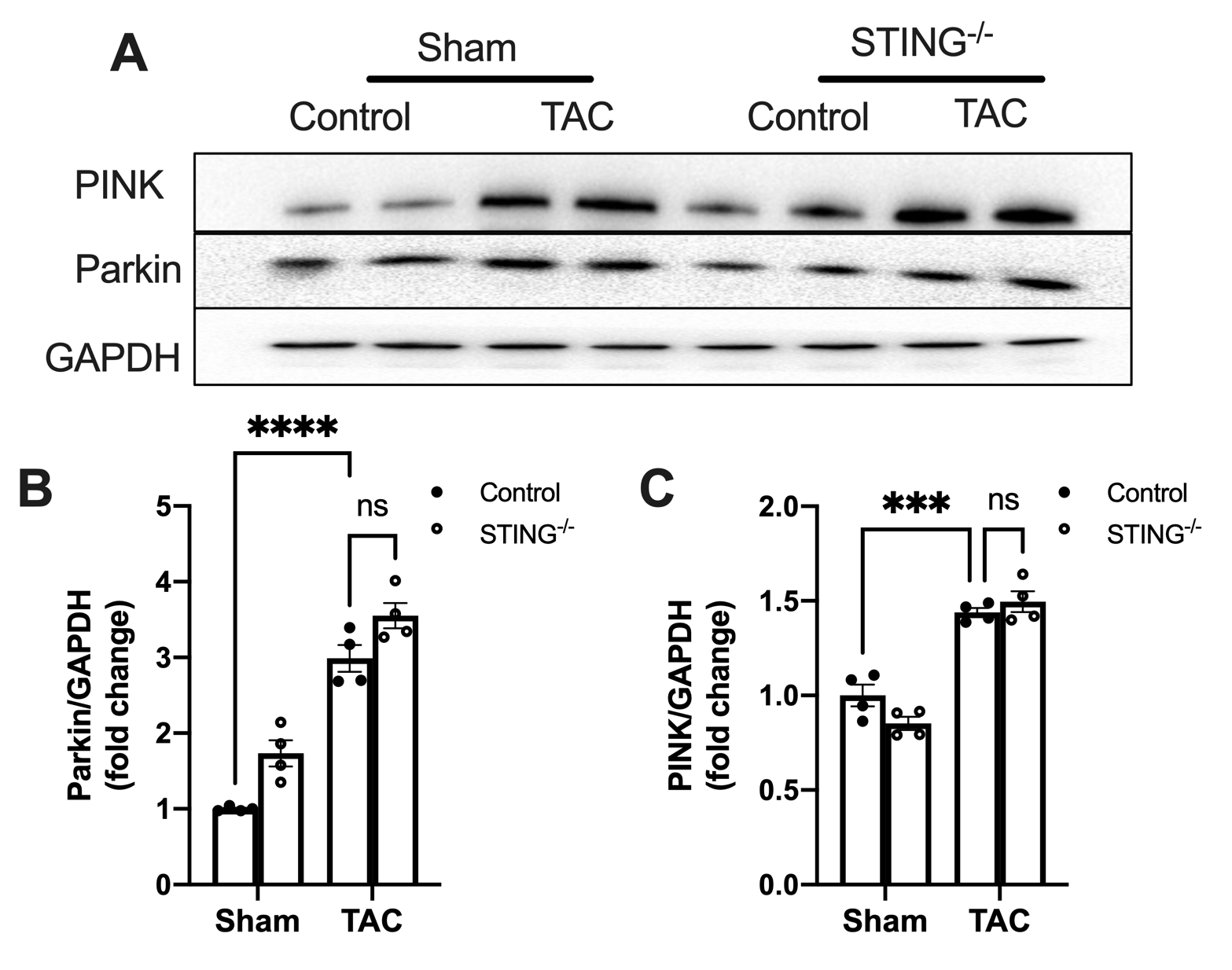


**Fig. S2** The effects of STING on the PINK and Parkin protein expression in TAC hearts. **(A–C)** Expression and quantitative results of PINK and Parkin in TAC heart with or without STING knockout (*n*=4). Data are presented as the mean ± SEM;***P*<0.01, ****P*<0.001,ns, no significant.


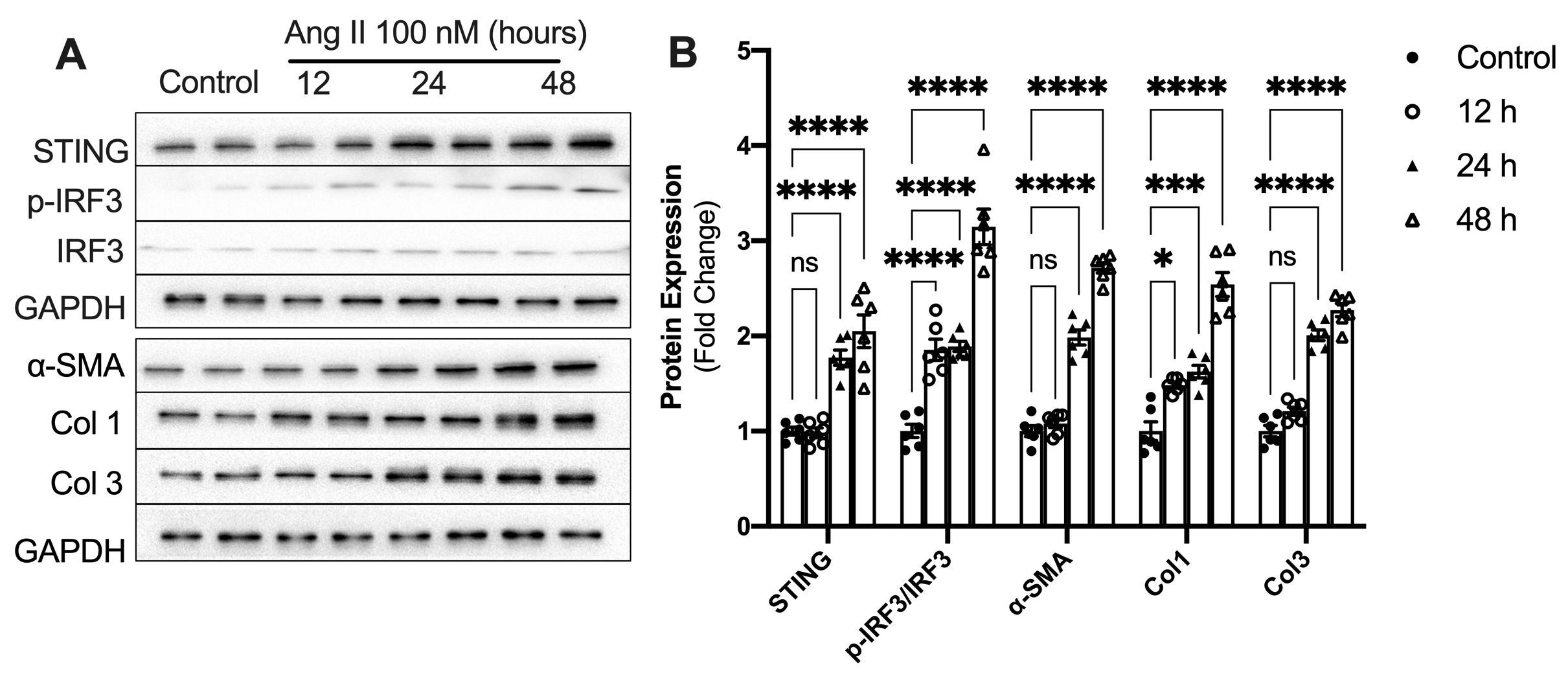


**Fig. S3** Ang II leads to STING activation and differentiation of CFs. **(A)** Protein levels of STING, p-IRF3, IRF3, α-SMA and collagen (type I and III) in CFs exposed to Ang II (100 nM) at different time points. The quantifications are shown in **(B)** (*n*=6). Data are presented as the mean ± SEM. ****P*<0.001, *****P*<0.0001, ns, no significant.. STING, stimulator of interferon genes. Ang II, angiotensin II. CFs, cardiac fibroblasts. IRF3, interferon regulatory factor 3. p-, phospho. α-SMA, α smooth muscle actin.


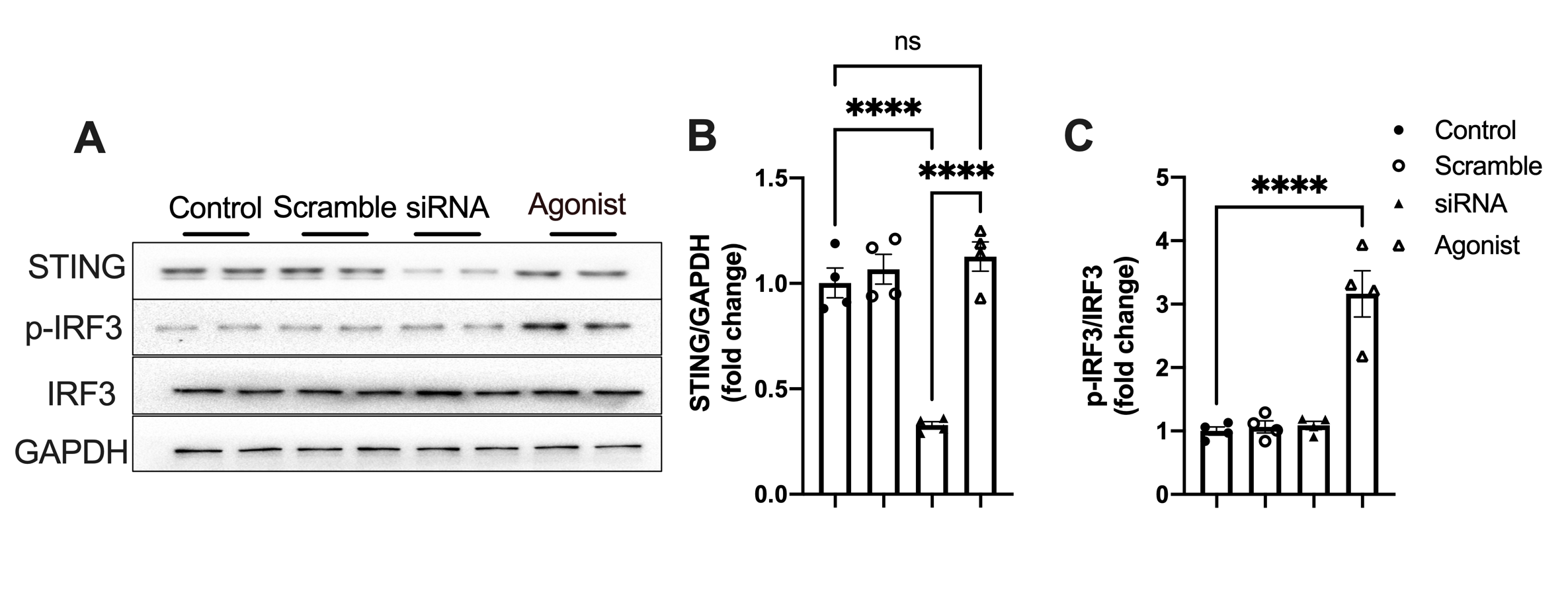


**Fig. S4** The effects of siRNA-STING and selective STING receptor agonist on the STING pathway in CFs. **(A–C)** Expression and quantitative results of STING, p-IRF3 and IRF3 in CFs treated with siRNA-STING or agonist (*n*=4). Data are presented as the mean ± SEM. *****P*<0.0001, ns, no significant. STING, stimulator of interferon genes. CFs, cardiac fibroblasts. IRF3, interferon regulatory factor 3. p-, phospho.


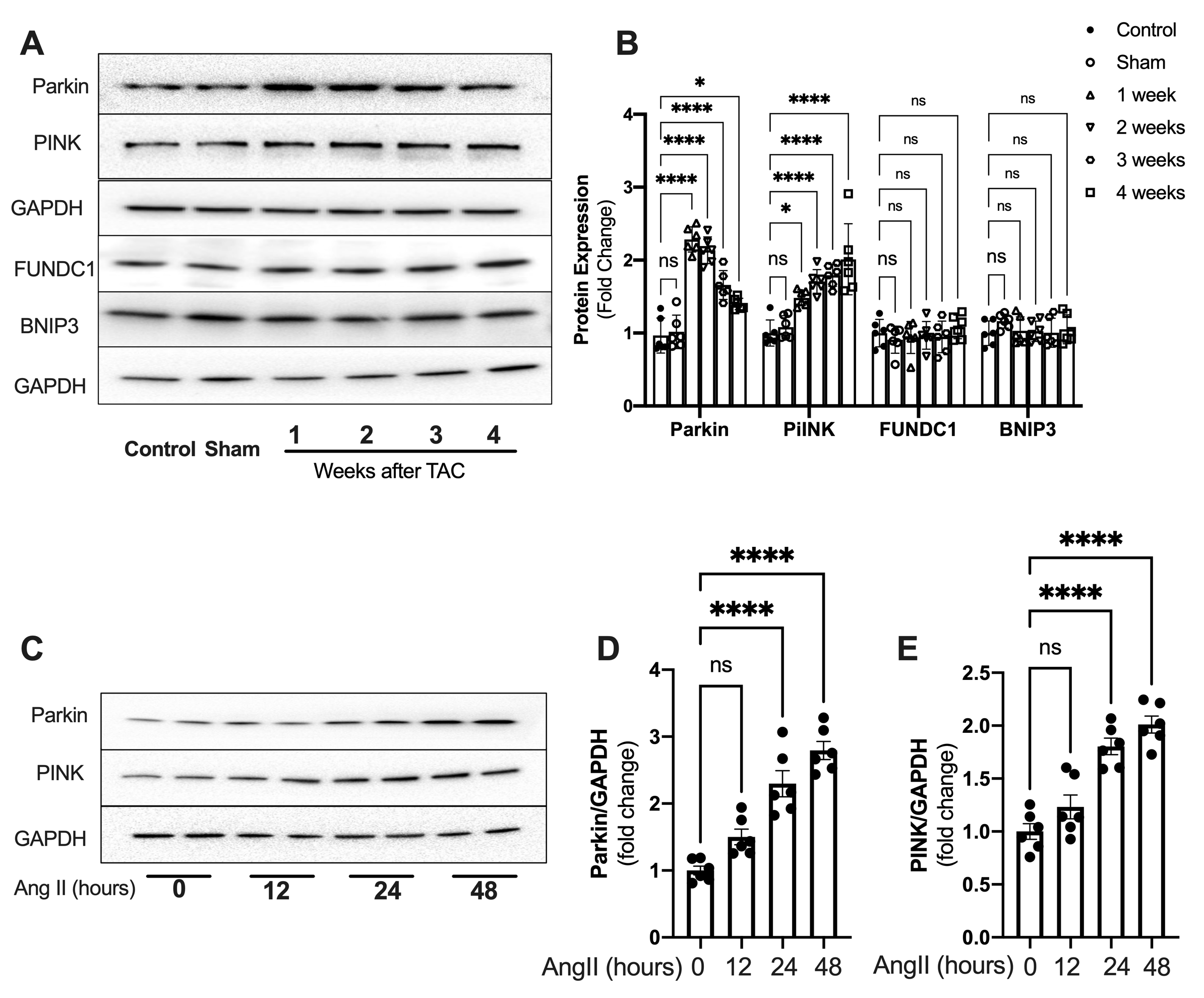


**Fig. S5** PINK-Parkin mitophggic pathway was activated in TAC hearts. **(A)** Protein levels of Parkin, PINK, FUNDC1 and BNIP3 in the hearts of TAC mice. Quantifications are shown in **(B)** (*n*=6). **(C)** Protein levels of Parkin and PINK in CFs exposed to Ang II (100 nM) at different time points. Quantifications are shown in **(D)** and **(E)** (*n*=6). Data are presented as the mean ± SEM. **P*<0.05, *****P*<0.0001, ns, no significant.


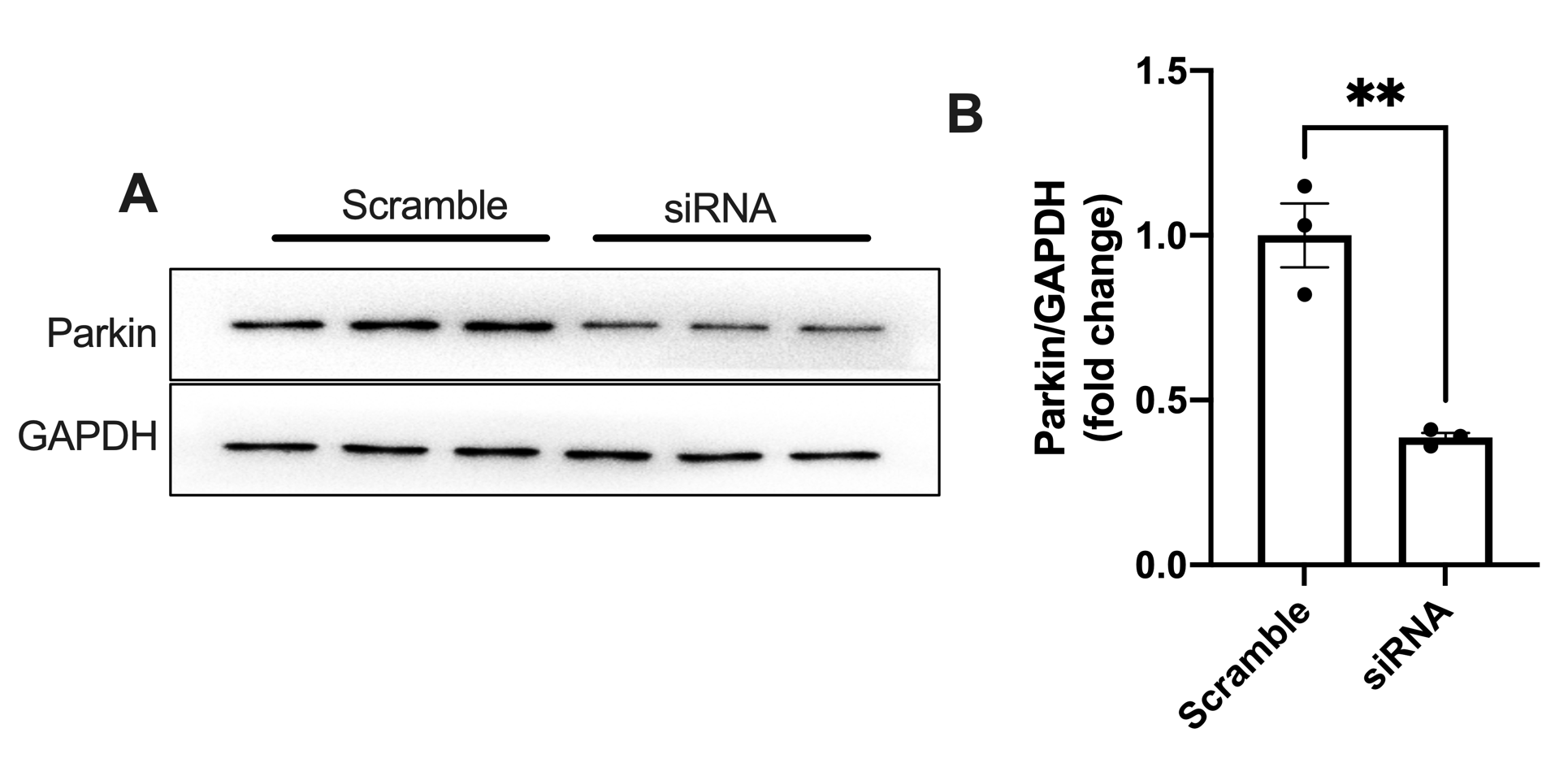


**Fig. S6** The effect of siRNA-mediated Parkin knockdown in CFs was assessed by western blotting. (*n*=3). ***P*<0.01. CFs, cardiac fibroblasts.


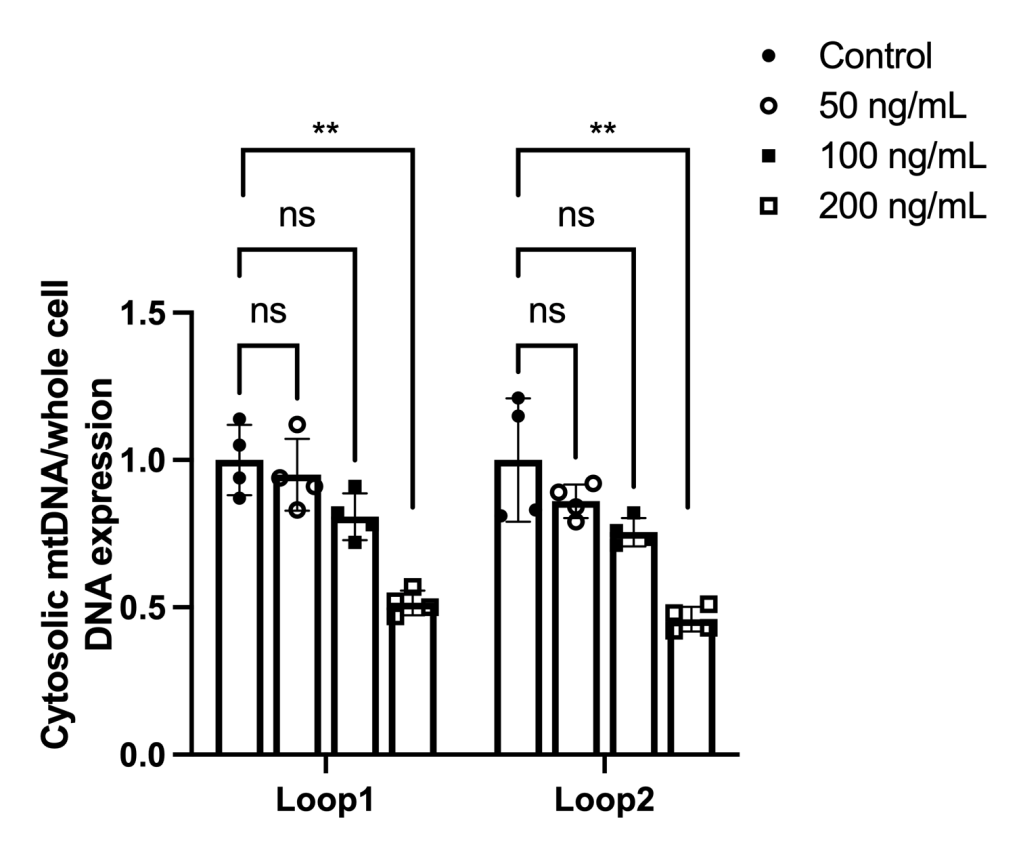


**Fig. S7** Cells were cultured with different concentrations of EtBr at 50, 100, or 200 ng/ml for 4 days. The efficacy of EtBr-mediated mtDNA depletion in CFs was assessed by western blotting. (*n*=4). ***P*<0.01ns, no significant. . CFs, cardiac fibroblasts. EtBr, ethidium bromide.

**Table S1** The used Real-time PCR primers in this study.

| Primers |  |  | Forward |  |  |  | Reverse |  |  |
| --- | --- | --- | --- | --- | --- | --- | --- | --- | --- |
| IFNβ | CTGCGTTCCTGCTGTGCTTCTCCA | | | | GAAGTCCGCCCTGTAGGTGAGGTTGA | | | | |
| Cxcl10 | TTTCTGCCTCATCCTGCTGGGTCTGA | | | | TGTGCGTGGCTTCACTCCAGTTAAGG | | | | |
| Ifit1 | AGGCTGGAGTGTGCTGAGATGGACTG | | | | TGTGCTGCTGAGGGCTTCTTCAATGT | | | | |
| Ifit2 | CAGAGGAAGAGGTTGCCTGGAGAGTG | | | | CTTGGTCAGGATGCTGTTGCTGGATG | | | | |
| Dloop1 | AATCTACCATCCTCCGTGAAACC | | | | TCAGTTTAGCTACCCCCAAGTTTAA | | | | |
| Dloop2 | CCCTTCCCCATTTGGTCT | | | | TGGTTTCACGGAGGATGG | | | | |
| GADPH | ATGGTGAAGGTCGGTGTGAACGGATT | | | | GTCTCGCTCCTGGAAGATGGTGATGG | | | | |
